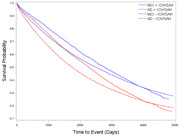# All‐cause mortality among veterans with mild cognitive impairment and Alzheimer’s dementia who have Intracerebral hemorrhage and subarachnoid hemorrhage

**DOI:** 10.1002/alz.089735

**Published:** 2025-01-09

**Authors:** Byron J. Aguilar, Ying Wang, Vanesa Carlota Andreu Arasa, Dan Berlowitz, Brant Mittler, Peter J Morin, Joel Reisman, Myriam Abdennadher, Henry W Querfurth, Raymond Zhang, Amir Abbas Tahami Monfared, Quanwu Zhang, Weiming Xia, Byron J. Aguilar

**Affiliations:** ^1^ The Bedford VA Research Corporation, Inc., Bedford, MA USA; ^2^ Geriatric Research Education & Clinical Center, VA Bedford Healthcare System, Bedford, MA USA; ^3^ Wentworth Institute of Technology, Boston, MA USA; ^4^ Boston University Chobanian & Avedisian School of Medicine, Boston, MA USA; ^5^ Neuroradiology Service, VA Boston Healthcare System, Boston, MA USA; ^6^ University of Massachusetts Lowell, Lowell, MA USA; ^7^ Geriatric Research Education & Clinical Center, VA South Texas Healthcare System, San Antonio, TX USA; ^8^ Center for Healthcare Organization & Implementation, VA Bedford Healthcare System, Bedford, MA USA; ^9^ Tufts Medical Center, Tufts University, Boton, MA USA; ^10^ Alzheimer’s Disease & Brain Health, Eisai Inc., Nutley, NJ USA; ^11^ McGill University, Montreal, QC Canada

## Abstract

**Background:**

Cerebral amyloid angiopathy (CAA) is a significant contributor to hemorrhagic stroke, notably lobar intracerebral hemorrhage (ICH) and convexity subarachnoid hemorrhage (SAH), both of which have been observed in patients with MCI/AD. To evaluate all‐cause mortality among veterans with mild cognitive impairment (MCI) and Alzheimer’s dementia (AD) with/without Intracerebral hemorrhage and subarachnoid hemorrhage (ICH/SAH) in the United States (US) Veterans Affairs Healthcare System (VAHS).

**Method:**

Veterans with MCI or AD were identified based on having clinical notes or diagnostic codes in the VAHS database (2010‐2019). ICH and SAH were identified with ICD‐10 codes I61.x and I60.x, respectively. Survival curves were generated, and Poisson regression was used to adjust for baseline characteristics.

**Result:**

A total of 853,791 veterans with MCI/AD were included, with a mean age of 74 years, of which 96% were male, 5% Hispanic, 15% Black, and 74% White; 32% had MCI and 68% had AD. Approximately 0.6% of the overall sample had ICH/SAH. The observed mortality rates per 1000 person‐years were 130 for males and 60 for females and 138 for AD vs 102 for MCI. Mortality rates for Veterans with MCI/AD with and without ICH was 102 and 127, and with and without SAH was 83 and 127, respectively. Kaplan‐Meier curves showed a higher survival probability for Veterans with ICH/SAH events vs those without the events (*P*<0.01) and for AD vs MCI (*P*< 0.01; **Figure**). The mortality risk was lower for MCI than AD overall (IRR = 0.83, *P*< 0.01). The death rate in veterans with MCI/AD was statistically significantly lower for those with vs without ICH/SAH even after adjustment (IRR = 0.85, p<0.01). Death rate was higher in non‐Hispanic vs Hispanic (IRR = 1.23, *P*<0.01) and for White vs Black veterans (IRR = 1.05, *P*<0.01).

**Conclusion:**

In US VAHS, AD was associated with an increased risk of death than MCI; ICH/SAH did not increase mortality risk. After adjustment, mortality rates were found to be 23% higher for non‐Hispanic vs Hispanic groups and 5% higher for White vs Black groups. Those findings will be further examined by incorporating Medicare data for Veterans who had dual eligibility for both VA and Medicare coverage.